# Tough PEGDA-Based Bioadhesive Hydrogels for High-Strength In Situ Cartilage Adhesion

**DOI:** 10.1021/acsomega.5c05254

**Published:** 2025-09-16

**Authors:** Kerem Veral, Rumeysa Tutar

**Affiliations:** Department of Chemistry, Faculty of Engineering, 532719 Istanbul University-Cerrahpaşa, Avcılar, Istanbul 34320, Turkey

## Abstract

Injuries to articular cartilage present considerable difficulty in musculoskeletal medicine owing to the tissue’s restricted regenerative capacity. This study sought to formulate and assess adhesive hydrogels to improve both adhesive and cohesive strengths for cartilage injuries. Injectable and in situ double-cross-linkable polyethylene glycol diacrylate (PEGDA)-based bioadhesive hydrogels aim to substantially improve the adhesion strength for the repair of cartilage injuries. PEGDA was prepared by adding acrylate groups to polyethylene glycol (PEG), whereas methacrylated alginate (AlgMA) was created by adding methacrylate groups to alginate. The PEG was altered to enhance its mechanical and adhesive characteristics. PEGDA was subsequently combined with AlgMA to enhance its cohesive characteristics. When the amine thiol in the cartilage tissue interacts with the PEGDA/AlgMA framework, it creates amide bonds and thioesters. The bioadhesives were carefully tested for their physical and chemical properties, how much they swell, how they break down over time, and how strong they are, including tests for pressure and how well they close wounds both in vitro and ex vivo. As a result, our improved hydrogel formulas showed ex vivo burst pressure of up to 194.5 ± 8.5 kPa and 335.5 ± 3.5 kPa lap shear, with wound closure strengths of 643.5 ± 46 kPa in vitro and 138 ± 8.5 kPa in tests ex vivo—numbers that are much higher than those of clinical fibrin adhesives and other similar materials. These results demonstrate that our composite hydrogel technology is a better bioactive adhesive for cartilage tissue sealing. Our findings will enhance the expanding research on nature-inspired biomaterials and pave the way for the clinical application of next-generation bioadhesives.

## Graphical abstract

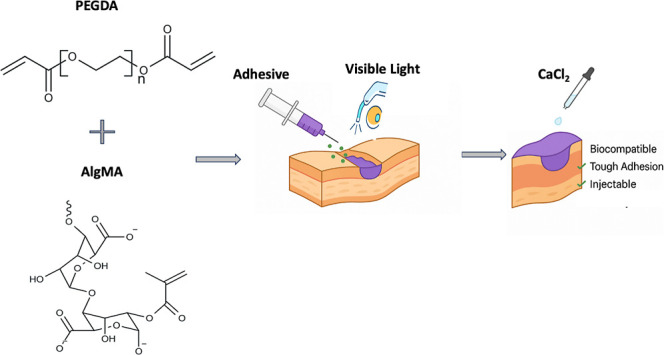

## 1 Introduction

Articular cartilage is a highly differentiated tissue with limited healing capacity. It receives nutrients from the synovial fluid. Therefore, because blood-derived growth factors cannot reach this tissue, repair or regeneration occurs within the tissue.^1^ Every year, one million surgeries are performed in the United States to repair cartilage injuries. Research on cartilage repair using cells, scaffolds, or signaling molecules, either alone or in combination, has become increasingly common in clinical practice. This field of research is known as tissue engineering.^2^ Articular cartilage possesses a restricted capacity for regeneration owing to its avascular characteristics, and the extracellular matrix (ECM) is comprised of collagen and proteoglycans. The lack of blood circulation hinders the transport of inflammatory mediators to the injured regions, therefore obstructing the natural healing process.^3,4^ Furthermore, ECM restricts cell movement.^5^

Peptide-based hydrogels are commonly used in tissue matrix development because of their superior biocompatibility compared with many other materials. Polyethylene glycol (PEG), a synthetic polymer approved by the FDA, has been employed in various biomedical applications. However, like many other polymers, PEG exhibits a complex interplay between its various properties, making it challenging to finely control its specific characteristics.^6,7^ Importantly, peptides can be acrylated and photopolymerized using acrylated PEG to create hydrogels.^8^ PEGDA is a derivative of PEG characterized by reactive acrylate groups at both termini, enabling the formation of hydrogels with superior mechanical characteristics via addition. Hydrogels based on PEGDA, which facilitate the adhesion and proliferation of chondrocytes and mesenchymal stem cells (MSCs), have been extensively studied for their potential in cartilage regeneration.^9–12^ Callahan et al. synthesized an arginine-glycine-aspartic acid (RGD) peptide and used a PEGDA hydrogel scaffold that was functionalized to enhance cell adhesion. Their findings suggest that the mechanical properties of these hydrogel scaffolds are essential for influencing the behavior of human osteoarthritic chondrocytes.^13^ Another study focused on the development of ChonDux hydrogel to address cartilage defects in microfracture surgery. This hydrogel system consisted of a PEG/HA network combined with chondroitin sulfate adhesive.^14^ In this study, polyethylene glycol (PEG) was first modified into PEG diacrylate (PEGDA) to enable photo-cross-linking within the hydrogel. Researchers have discovered that combining HA with PEGDA promotes the chondrogenesis of mesenchymal stem cells in vivo.^15^

Alginate methacrylate (AlgMA) hydrogels are frequently used in biomedical applications because of their ability to undergo photo-cross-linking. This property not only enables the creation of cell-laden hydrogels but also helps in fine-tuning physical attributes such as mechanical properties, pore size distribution, and degradation rate. In the past decade, various modifications have been made to AlgMA hydrogels to improve their physicochemical and biological properties for tissue-engineering applications. One study showed that a hybrid system composed of methacrylated PEG (PEGMA) and oxidized methacrylated alginate allowed for the examination of cellular interactions and microenvironments in complex tissues using stereolithography to create 3D spatially patterned cocultures of different cell types.^16^ In another study, oxidized methacrylated alginate was used as a cross-linking agent within methacrylated PEG (PEGMA) and poly­(*N*-hydroxyethyl acrylamide) polymer networks, enhancing the durability and stiffness of the hydrogels compared with those using short-chain cross-linkers. Interestingly, modifying the oxidation level of methacrylated alginate was found to control the biodegradation rate of the composite, while preserving its mechanical properties.^6^

The aforementioned studies focused primarily on using AlgMA as an adjunct to enhance the physical properties of PEG. In contrast, some researchers have attempted to establish a secondary cross-linking network by incorporating specific PEG derivatives into AlgMA-based hydrogels.^17,18^ Currently, PEG-based materials are widely utilized in various medical applications owing to their biocompatibility and versatility. Prominent applications include FocalSeal,^19,20^ CoSeal,^21^ Adherus, SprayGel, and OcuSeal.^22^ The widespread adoption of PEG in these applications highlights its critical role in advancing surgical techniques and improving patient outcomes through enhanced adhesion, sealing, and hemostatic capabilities.

Most studies have focused on repair of cartilage defects.^23–25^ Dually cross-linked hydrogels were fabricated using hyaluronic acid and chitosan for articular cartilage damages.^23^ In another study, methacrylate-grafted hyaluronic acid (HA) was used to prepare a versatile hybrid photo-cross-linkable (HPC) hydrogel.^24^ In Another study, alginate-dopamine, chondroitin sulfate, and regenerated silk fibroin (AD/CS/RSF) were used to create a cross-linked network. Standard tissue adhesion protocols from the American Society for Testing and Materials (ASTM) were used to determine the adhesive properties of the hydrogels. The development of an adhesion mechanism for cartilage injuries in the presence of engineered bioadhesive hydrogels was demonstrated through the implementation of diverse in vitro and ex vivo experimental protocols. These methodologies elucidated the underlying interactions and bonding efficacy between hydrogels and cartilage tissue, providing critical insights into the functional performance and potential therapeutic applications of such bioadhesive systems in cartilage repair. In vitro and ex vivo lap-shear and wound closure test configurations have been employed to evaluate the interfacial strength associated with various cartilage bonding techniques such as integrative repair, chemical cross-linking, and the application of adhesives. This method provides a quantitative assessment of the bonding efficacy, facilitating the comparison of different strategies aimed at enhancing cartilage repair and integration.

Although current biomaterials and tissue engineering approaches have demonstrated encouraging results in the management of full-thickness articular cartilage abnormalities, the treatment of partial-thickness lesions continues to pose a clinical challenge, particularly in the early to midstages of osteoarthritis. This study involved the development and characterization of synthetic bioadhesive hydrogels to facilitate their successful integration with native human cartilage tissue. The formulation integrated the mechanical durability, biocompatibility, and inherent adhesion characteristics of PEGDA with the elasticity and improved the adhesive capabilities of ALGMA. The tissue adhesion efficacy of the resultant composite hydrogels was methodically assessed using ASTM-standardized lap shear and burst pressure testing.

## 2 Materials and Methods

### 2.1 Materials

Sodium alginate (Na-alginate), methacrylic anhydride (MA, purity Z94%), triethanolamine (TEA, purity Z99%), N-vinylcaprolactam (VC; purity Z98%), Eosin Y, calcium chloride dihydrate (purity Z99%), poly­(ethylene glycol) (Average Mn 6000), triethylamine (purity ≥ 99.5%), hexane (purity ≥ 97%), and acryloyl chloride were purchased from Sigma-Aldrich. Benzene was purchased from AnalaR, BDH. Dulbecco’s phosphate-buffered saline (DPBS) and Dulbecco’s modified Eagle’s medium (DMEM) were purchased from Thermo Fisher Scientific and were manufactured by GIBCO/Life Technologies. Fetal bovine serum (FBS, HyClone, SH30088.03), GlutaMAX (Gibco, 35050061), HEPES (HyClone, SH30237.01), Roche LDH Cytotoxicity Kit (Roche, 11644793001), and penicillin/streptomycin (Capricorn; PS-B). L929 cells.

### 2.2 Synthesis of PEGDA

PEGDA was prepared according to a previously reported method.^26^ In summary, 12 g of PEG (6000 Da) was fully dissolved in 150 mL of benzene at 45 °C while stirring. The transparent solution was allowed to cool to the ambient temperature. Subsequently, 12.0 mmol of triethylamine and acryloyl chloride acrylate (equivalent amount) were added dropwise to the PEG solution. The mixture was agitated under nitrogen at 80 °C for 3 h, at which time the reaction occurred. The solution was filtered through standard filter paper to exclude the insoluble triethylamine salts produced during the process. Concurrently, 700 mL hexane was cooled to 4 °C. The PEGDA product was precipitated with chilled hexane. The PEGDA polymer was subsequently dried at ambient temperature for 24 h and preserved at approximately 4 °C until further use.

### 2.3 Synthesis of AlgMA

AlgMA was methacrylated as previously described.^27^ Sodium alginate at a concentration of 2.5% (w/v) was measured and thoroughly dissolved in 100 mL of deionized water at ambient temperature while stirring at 250 rpm. Subsequently, MA (37.5 mL) of MA was added incrementally to the produced alginate solution. The pH of the liquid was adjusted to 7 using a 5N NaOH solution. The reaction was continued for 72 h. AlgMA was extracted from the solution by using ethanol. The AlgMA solution was then transferred to ethanol (200 mL). AlgMA was collected on a filter paper. The precipitate was subsequently dissolved in 100 mL of deionized water. The combination was subsequently dialyzed against deionized (DI) water with 12–14 kDa dialysis tubing for 1 week to eliminate contaminants, including methacrylic acid. The purified solution was lyophilized in a freeze-dryer to produce white foam.

### 2.4 Preparation of Tissue-Adhesive Hydrogel

To prepare the prepolymer solution, 20% w/v PEGDA was prepared in response to different concentrations of AlgMA (2, 4, and 6% w/v). Briefly, PEGDA was dissolved in photoinitiator solutions prepared with visible light photoinitiators TEA, 1.875% (w/v), VC 1.25% (w/v), and Eosin Y disodium salt (0.5 mM) for 10 min at 50 °C. AlgMA (2%, 4%, and 6% w/v) products were weighed and added to the prepared 10% w/v PEGDA solutions. After heating, the precursor solution was cooled to room temperature before further use, ensuring its applicability in in situ clinical settings without additional heating requirements. To prepare the hydrogel, 100 μL of the prepolymer solutions were taken from the prepared precursor solutions and pipetted into the prepared PDMS molds. The prepolymer solutions were exposed to visible light (blue–green light (450–550 nm) at 100 mW/cm) using a small LED light source. Visible light exposure times were optimized to 240 s to make the hydrogels. Following the formation of the chemically cross-linked structure, the hydrogel was incubated in a 1 M Ca^2+^ solution for 5 min to achieve ionic cross-linking.

### 2.5 Chemical and Morphological Characterization

Chemical characterization of biopolymers and cross-linked hydrogels was conducted using ATR-FTIR and ^1^H NMR techniques. The ATR-FTIR spectra of all the examined substances were obtained in the range of 4000 to 500 cm-1 using a Jasco FT/IR 6700 spectrophotometer. ^1^H NMR spectra of the polymers were obtained using a JEOL ECZ500R (11.75 T) spectrometer in conjunction with a high-performance UltrashieldTM 500 MHz superconducting magnet. Degree of methacrylation (DoM) was calculated according to the ratio of methacryl groups to monomer units in the alginate backbone for AlgMA (eq 1). The divided by 2 is due to the 2 protons of the vinyl group.

1 $$\left(DoM\%\right)=\frac{Integral\;of\;methacrylate\;vinyl\;protons\;\left(5.65-6.1\;ppm\right)}{Integral\;of\;reference\;sugar\;proton\;\left(3.5-4.0\;ppm\right)}\times \left(\frac{1}{2}\right)\times 100$$

Sixteen scans were performed to determine the average signal-to-noise ratio. D2O was used as the solvent. Scanning electron microscopy (SEM; ESEM-FEG, Quanta FEG 650 ESEM, FEI Inc.) was used to examine the topography and composition of the tissue matrices.

### 2.6 Physical Characterization

The Swelling Characteristics of the hybrid hydrogels were determined. First, 100 μL of the precursor solution (PEGDA/AlgMA) was pipetted into a PDMS (diameter = 8 mm and thickness = 1.5 mm) mold to prepare cross-linked (visible light/visible light and ionic) hydrogels. The cross-linked hydrogels were then frozen. Frozen hydrogels were lyophilized. Dried samples were weighed (Wi) and recorded. Next, the weighed samples were incubated in DPBS for 1, 3, 5, 7, 24, and 48 h at 37 °C to reach an equilibrium swelling state. Samples were then removed under different time conditions (*n* = 3) from the DPBS, and the swollen (Wf) hydrogel sample weights were measured. The swelling ratio was calculated using eq 2 for each material.^28^

The Degradation Characteristics were evaluated to determine the hydrogel weight loss. Briefly, the prepared solutions were cross-linked as described in the swelling characterization studies. The lyophilized materials were weighed (Wi) and incubated in DPBS solution containing 10 μg mL^–1^ collagenase at 37 °C for 1, 3, 5, 7, 24, 48 h and1, 2, and 3 weeks. Samples were then removed at different time points (*n* = 3) from DPBS, frozen, and lyophilized. The lyophilized materials were then reweighed (Wf) and recorded. The percent mass loss was calculated using eq 3.

2 $$Swelling\;\left(\%\right)=\frac{Wf-Wi}{Wi}\times 100$$

3 $$Lost\;weight\;\left(\%\right)=\frac{Wi-Wf}{Wi}\times 100$$

### 2.7 Compressive Strength Tests

Compressive stress–strain measurements were performed using a universal material testing machine (TA.XT plus Texture Analysis mechanical tester). For these tests, the hydrogels were cast in cylindrical molds with a diameter of 10 mm and depth of 5 mm. Prior to testing, samples were incubated in PBS at 37 °C for 1 h. During testing, the samples were compressed at a strain rate of 1 mm/min until failure. The compressive strengths of the samples were determined from the pressure recorded at the failure point. The compressive modulus was calculated by linear fitting of the stress–strain curve within a strain range of 10–15%.

### 2.8 In Vitro Tissue Sealing Characterization

#### 2.8.1 Burst Pressure

The sealing performance of the adhesive was assessed using thin collagen membranes as model substrates, following the protocol defined in ASTM F2392–04,^29^ to evaluate the burst strength of surgical sealant materials. First, wounds were simulated by damaging the collagen sheets using a punch. Subsequently, the prepared tissue adhesive solution was carefully applied to the damaged areas. To ensure complete cross-linking of the adhesive, light in the 450–550 nm wavelength range was applied using a VALO device for 240 s. After the curing process was completed, the membranes were placed between the two metal plates and tightly secured to ensure a sealed setup. The test system was equipped with a pump system and pressure measurement device connected to a computer. Once the experiment was initiated, pressure was applied gradually to the simulated wound using a pump system. During this process, the pressure at which the wound began to leak was recorded on a computer screen. This value was reported as the burst pressure of tissue adhesive.

#### 2.8.2 Lap–Shear Test

A setup compliant with the ASTM F2255–05 standard^30^ was used to evaluate the adhesion strength and resistance of the tissue adhesive to the shear stress. First, substrates (20% gelatin solution covered glass coverslips) were prepared according to the standard dimensions. The substrates were typically cut to a width of 25 mm and length of 76 mm, and their thicknesses were adjusted based on the material used. Subsequently, the prepared tissue adhesive was carefully applied to a 5–12 mm overlap region between the two substrates. Following the application, the adhesive was cured using light of 450–550 nm wavelength with a VALO device, and this process was performed for a specific duration (e.g., 240 s). During the testing phase, the specimens were aligned along a parallel axis using a tensile testing machine and clamped securely. The testing device applied a steady tensile force at low speed until the bonded area failed. The maximum force applied at the failure point was recorded. The recorded value was calculated as shear strength, which represents the adhesion strength of the tissue adhesive. This calculation was performed by dividing the applied force (F) by the bonded area (A). The results were compared with those of other adhesives reported in the literature (e.g., fibrin adhesives) to evaluate the adhesion capacity of the tissue adhesive and its suitability for cartilage application.

#### 2.8.3 Wound Closure Strength

The wound closure strength of the tissue adhesive was evaluated according to ASTM F2458–05 standard.^31^ First, the substrates (collagen sheets) were prepared according to standard dimensions. The substrates were typically cut to a width of 25 mm and length of 76 mm, and their thicknesses were adjusted based on the material used. A simulated wound was then created on the substrates, typically–5–10 mm in diameter. Subsequently, the prepared tissue adhesive was carefully applied to the damaged area. To ensure full cross-linking of the adhesive, light of 450–550 nm wavelength was applied using a VALO device, and this process was carried out for a specific duration (e.g., 240 s). During the test phase, the specimens were placed parallel to the axis in a tensile testing machine and securely clamped. The testing device applied a steady tensile force at low speed. The force was gradually increased until the bonded area failed and the maximum force applied at the failure point was recorded. The recorded maximum force was considered the wound closure strength of the tissue adhesive. This value was calculated by dividing the applied force (F) by the bonded area (A). The results were compared with those of other adhesives reported in the literature (e.g., fibrin adhesives) to assess the adhesive capacity of the tissue adhesive and its suitability for cartilage application.

### 2.9 Ex Vivo Tissue Sealing Characterization

#### 2.9.1 Burst Pressure

An ex vivo burst pressure test was performed using a cartilage sample obtained from the articular surface of the shoulder joint of a sheep. Specifically, hyaline cartilage (Cartilago hyalina) was carefully dissected from the glenoid cavity of the scapula and the head of the humerus. These tissues were collected post-mortem from animals that had died due to unrelated causes. No live animals were sacrificed specifically for this study, and thus animal ethics approval was not required. At the start of the test, damage was induced in the cartilage sample by using a punch to simulate a wound. The prepared tissue adhesive solution was then carefully applied to the damaged area, and after application, the adhesive was cross-linked using a VALO device with light exposure in the 450–550 nm wavelength range for 240 s. In the test setup, the membranes were tightly placed between the two metal plates to ensure sealing. The system was equipped with a pump and pressure measurement device connected to a computer. Once the test began, increasing pressure was applied to the simulated wound through the pump system. The pressure at which the wound began to leak was recorded on the computer screen. The obtained value is reported as the burst pressure of the tissue adhesive.

#### 2.9.2 Wound Closure Strength

The wound closure strength of the tissue adhesive was evaluated according to the ASTM F2458–05 standard. First, a cartilage sample (hyaline cartilage) obtained from sheep was prepared according to the standard dimensions. A simulated wound was created on the cartilage surface by using a punch. The prepared tissue adhesive solution was then carefully applied to the damaged area, and after application, the adhesive was cross-linked using a VALO device with light exposure in the 450–550 nm wavelength range for 240 s. During the test phase, the specimens were placed parallel to the axis in a tensile testing machine and securely clamped. The testing device applied a steady tensile force at low speed. The force was gradually increased until the bonded area failed and the maximum force applied at the failure point was recorded. The recorded maximum force represented the wound closure strength of the tissue adhesive. This value was calculated by dividing the applied force (F) by the bonded area (A).

#### 2.9.3 Lap–Shear Test

An ex vivo lap shear test was performed using a cartilage sample obtained from the articular surface of the shoulder joint of a sheep. Specifically, hyaline cartilage (Cartilago hyalina) was carefully dissected from the glenoid cavity of the scapula and the head of the humerus. To begin the test, the cartilage substrates were cut into standard dimensions, typically 25 mm in width and 76 mm in length, with the thickness adjusted based on the material. The prepared tissue adhesive was applied to the surfaces of cartilage samples with a controlled overlap of 5–12 mm. After application, the adhesive was cross-linked using a VALO device with light exposure in the 450–550 nm wavelength range for 240 s, ensuring proper curing. The substrates were then placed in a tensile testing machine along a parallel axis and securely clamped. A constant tensile force was applied at a low speed, which gradually increased until failure occurred in the adhesive bond. The maximum force applied during the failure was recorded. The maximum force value obtained was calculated as the shear strength, which represents the mechanical strength of the adhesive. This calculation was performed by dividing the applied force (F) by the adhesive-bonding area (A). This test was conducted to evaluate the ability of the adhesive to maintain the structural integrity of cartilage under tensile stress.

### 2.10 In Vitro Cytocompatibility Tests

The controls used in the study were defined as medium control (Cm), live control (Fibroblast), dead control (C0), active substance control, cross-reaction control, and experimental samples. Only cell-free medium was used as the medium control, cells and medium were used as the live control, and cell suspension containing 2% Triton-X was used as the dead control. While the active substance control allowed the tested material to be evaluated without interacting with the cells, the active substance and the LDH standard were applied together in the cross-reaction control. In the experimental samples, the active material was used together with the cells, and evaluation was performed. Cells stored at −150 °C were thawed in a water bath at 37 °C for 2 min and then immediately transferred to a medium containing five times its volume of FBS. The suspension was centrifuged at 300*g* for 5 min to sediment the cells. The supernatant of the resulting cell pellet was carefully removed, and the remaining cells were slowly and homogeneously suspended in complete medium. The cells were seeded into culture flasks at a density of 10^4^ cells/cm^2^. The complete medium used in this process contained 90% basal medium, 10% heat-inactivated FBS, 1% HEPES, 1% glutamine, and 1% antibiotic (Pen-Strep). After the cells were multiplied for two passages, they were seeded into 96-well cell culture plates at 10,000 cells per well, and experimental studies were initiated.

## 3 Results and Discussion

### 3.1 Molecular and Chemical Characterizations

The molecular structure modeling of the hydrogels is shown in Figure 1a. Acrylate and methacrylate modifications were applied to PEG and Na-alginate, respectively, to improve the adhesion mechanism. Double cross-linkable hydrogels were designed to enhance both the adhesive and cohesive strengths. The bonding structures of tissue adhesives were designed to strengthen their interactions. Different types of interactions were observed for different bonding strengths (kJ/mol). Visible light was used to make the adhesives cross-linkable. Covalent bonding with a bonding strength of 800 kJ/mol was created using acrylate and methacrylate interactions. In addition, based on functional groups, such as acrylic acid, hydrogen bonding was observed with a bonding strength of 65 kJ/mol. Moreover, electrostatic bonds were created due to AlgMA-Ca^2+^ interactions with 25 kj/mol bonding strength. The adsorption kinetics and diffusion behavior of the adhesive at the interface affected the electrostatic interactions.

**Figure 1:**
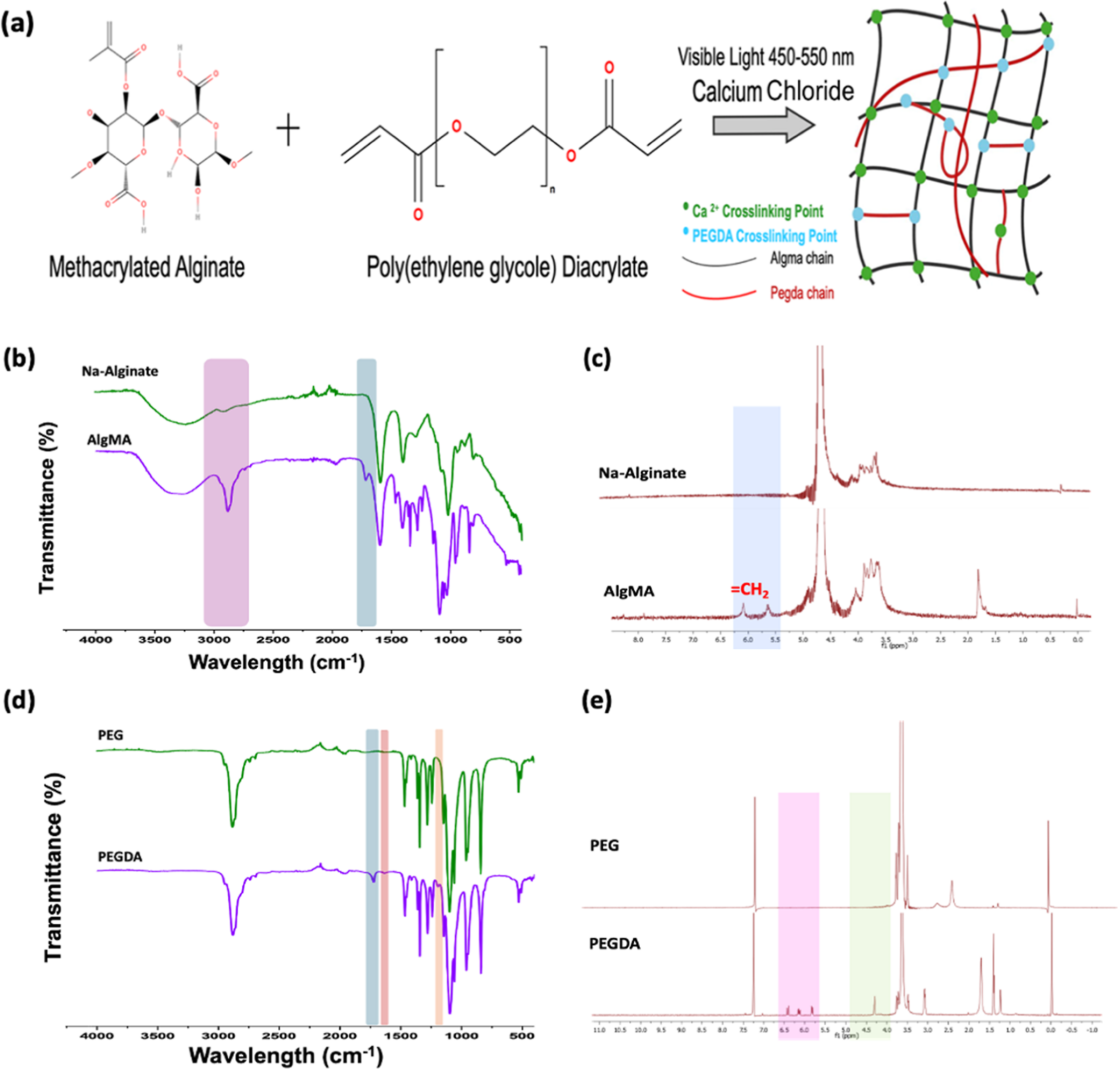
(a) Molecular structure modeling of hydrogels. (b) FTIR-ATR and (c) NMR analyses of Na-alginate and AlgMA. (d) FTIR-ATR and (e) NMR analysis of PEG and PEGDA.

FTIR and ^1^H NMR were used to characterize the pristine Na-alginate, AlgMA, PEG, and PEGDA. Figure 1b–d show the FTIR-ATR spectra and Figure 1c–e show the NMR spectra of the synthesized chemical structures under optimized conditions.

The FTIR spectra of all examined biological materials were obtained in the range of 4000–500 cm^–1^ using a Jasco FT/IR 6700 spectrophotometer. The FTIR measurements validated the functionalization of the polymers and their associated chemical groups. The bands at 3700 to 3000 cm^–1^ and 2980 to 2850 cm^–1^ correspond to the stretching vibrations of the – OH and –CH groups, respectively.^32^ The graph illustrates aliphatic chains (2980–2850 cm^–1^), C=C aromatic structures (1600 cm^–1^), and carboxyl esters (1705 cm^–1^) derived from grafted methacrylic anhydride groups. The AlgMA spectrum has a shoulder about 1705 cm^–1^, indicating the enhancement of carboxylate anion (COO^–^) stretching bands (Figure 1b).^27,32,33^ The end-capping of PEG with acrylate or methacrylate is evident in the FTIR spectra (Figure 1d). The acrylated and methacrylated samples exhibit reduced absorbance intensity near 3400 cm^–1^ due to this functionalization, indicating that the photo-cross-linkable moiety is attached to the hydroxyl terminal of the polymers. The three distinct peaks at 1638, 1283, and 1110–1041 cm^–1^ signify the establishment of ester bonds between the acrylate or methacrylate groups and polymers. The C=O stretching frequency at 1638 cm^–1^ indicates the existence of α,β-unsaturated esters, specifically acrylate or methacrylate groups.^34^ The NMR spectrum shown in Figure 1c reveals characteristic peaks of the saccharide units in the Na-alginate backbone, occurring between 3.25 and 5.25 ppm.^35^ We can also observe that the peaks at 5.65 and 6.1 ppm for the methacrylate group of AlgMA and the methyl protons of methacrylate was observed at 1.8 ppm^34^. Peaks for methylene (δ5.3–5.8 ppm), methyl (δ1.8 ppm), and alginate (δ3.5–4 ppm) were observed in the spectra.^36^ The degree of methacrylation (DoM) was calculated 15%.

Functionalization of PEG was also verified by ^1^H NMR spectroscopy. Figure 1d illustrates that the protons of PEG acrylate were detected at 5.8 ppm and within the range of 6.1 to 6.4 ppm.^34^

### 3.2 Morphological and Physical Characterizations

Porous scaffolds have been used extensively for cartilage tissue regeneration.^37^ As shown in Figure 2b, as the amount of AlgMA in the hydrogel increases, the formation of porous structures also increases. Pores began to form even at 2% (w/v) AlgMA concentration. The hydrogel containing 2% (w/v) AlgMA exhibited smaller and more irregular pores, with an average pore size of 11 μm. This irregularity indicates a less structured network, which may lead to less consistent interactions with the surrounding tissue. Smaller pores can contribute to faster diffusion of nutrients and waste but could also limit the ability of the material to support cell infiltration and tissue growth. The hydrogel with 4% (w/v) AlgMA displayed the most regular and rounded pore structure with an average pore size of 35 μm. This uniformity suggests a more organized porous network, potentially offering better mechanical stability and controlled cell infiltration. Larger pores allow for a more efficient nutrient and oxygen exchange, which can facilitate tissue regeneration. The regularity of the pores also indicates the suitability of the material for supporting cell adhesion and growth in applications that require precise biological interactions.

**Figure 2:**
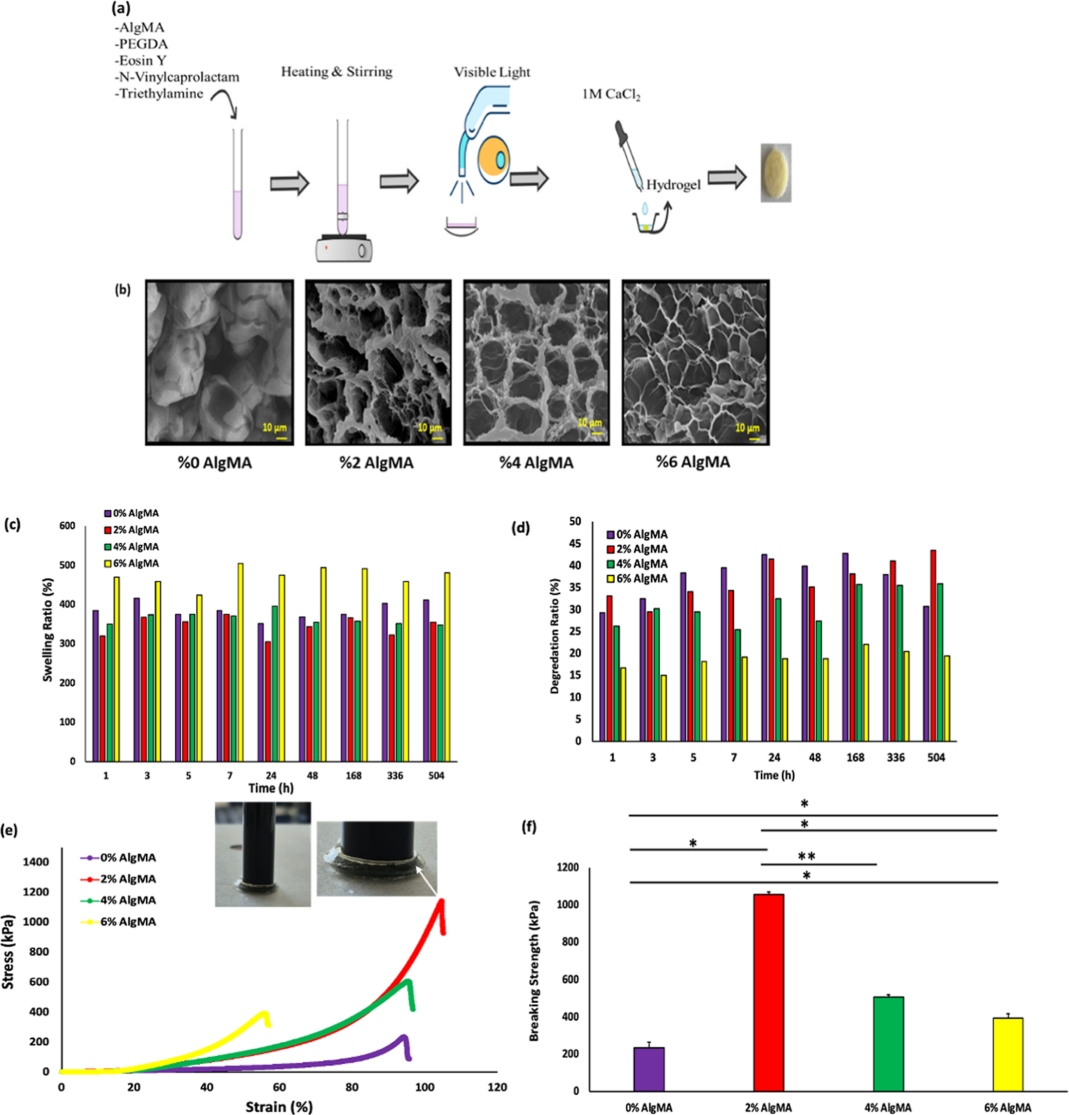
Characterization of physical and mechanical properties of hydrogels. (a) Rapid gelation of hydrogel prepolymer solution under visible light and Ca^2+^. (b) SEM images of the 0% (w/v) AlgMA, 2% (w/v) AlgMA, 4% (w/v) AlgMA, and 6% (w/v) AlgMA hydrogels. (c) Swelling ratio of different hydrogels over time at 37 °C in PBS solution. (d) Degradation ratios of different hydrogels in PBS with 10 μg mL^–1^ collagenase at 37 °C. (e) Compressive stress–strain curves of different composite hydrogels. (f) Compressive modulus (Breaking Strength) of different hydrogels (mean ± SD, *n* = 4).

The pore sizes were measured using ImageJ software to analyze the SEM images. The 6% (w/v) AlgMA hydrogel exhibited a structure resembling rounded pores, but with a more irregular distribution and an average pore size of 17 μm. Although slightly more irregular than the 4% (w/v) AlgMA hydrogel, this structure still presents potential for cell interaction, although it has a more variable pore distribution. The intermediate pore size may offer a balance between mechanical stability and cellular infiltration depending on the specific application.

In summary, the observed pore characteristics of these hydrogels, ranging from irregular and smaller pores in the 2% (w/v) AlgMA hydrogel to more regular and larger pores in the 4% (w/v) AlgMA hydrogel, are indicative of their potential mechanical properties and suitability for tissue-engineering applications. The size and uniformity of the pores can influence the mechanical strength, cell behavior, and overall performance of the hydrogel in supporting tissue regeneration.

As the PEGDA structures were photoactivated, the degree of cross-linking directly affected the swelling properties (Figure 2c). The control PEGDA hydrogel, which did not contain AlgMA, showed less swelling than hydrogels containing 6% (w/v) AlgMA. The cross-linking points enhance the strength of the PEGDA hydrogel. Notably, hydrogels containing 6% (w/v) AlgMA exhibited significant degradation resistance (Figure 2d) compared to other hybrid structures. Fibrin glue, commonly utilized as a tissue adhesive for cartilage injuries owing to its biocompatibility and efficacy in facilitating healing, undergoes enzymatic breakdown, which can be regulated by adjusting the fibrinogen concentration. Under in vivo settings, a fibrinogen concentration of 80 mg/mL resulted in the fibrin glue losing approximately 50% of its initial mass after 2 weeks and around 80% by five to 6 weeks.^38^ Most diseased cartilage is filled with healthy cartilage or other tissues, which generally require 6–8 months.^39^ Indeed, the precise degradation time is likely to vary and is influenced by numerous factors such as the type of cartilage (thickened or dense), cartilage thickness, age, vitamin D levels in the body, severity of the injury, and therapeutic outcome.^40^ Cartilage tissue can heal within 6–12 weeks, but the healing process can extend for up to 8 months. The tissue adhesive we developed, through various modifications, is designed to be optimized for the healing process and has shown promising results. In addition, degradation typically leads to a loss of mechanical strength and weakens the long-term stability of hydrogel mechanics.^41^ As the AlgMA concentration increased, the degradation rate of the composite structures decreased, resulting in an enhanced stability of the mechanical properties of the prepared tissue adhesives.

### 3.3 Mechanical Characterization

Compressive tests were performed on the as-prepared hydrogels to test their mechanical properties. As shown in Figure 2e,f, hydrogel disks were prepared by cross-linking in the presence of visible light and ions. In compression tests, a hydrogel sample with a 2% w/v AlgMA polymer content exhibited a maximum strain of approximately 105%. This unusually high strain is due to the soft, viscoelastic nature of the hydrogel and the strong interfacial adhesion between the sample and compression plates. This resulted in a ’barreling’ effect, characterized by the material bulging outward from its base and swelling at the edges (Figure 2e). This type of deformation is commonly observed in soft hydrogel systems, particularly when frictionless testing conditions are not met, and can lead to an overestimation of the true axial strain.

### 3.4 In Vitro Tissue-Sealing Characterization

ASTM standard tissue adhesion protocols were used to determine the adhesive properties of hydrogels.

#### 3.4.1 Burst Pressure

The in vitro burst pressure setup is illustrated in Figure 3a. An artificial hole was created using a medical punch (3 mm) to mimic a wound. Pregel solutions containing PEGDA (20% w/v) and different concentrations of AlgMA were pipetted into the wound area to seal artificial holes. First, visible light was applied to chemically cross-link the biadhesive hydrogel. Ionic cross-linking was performed in the presence of Ca^2+^.

**Figure 3:**
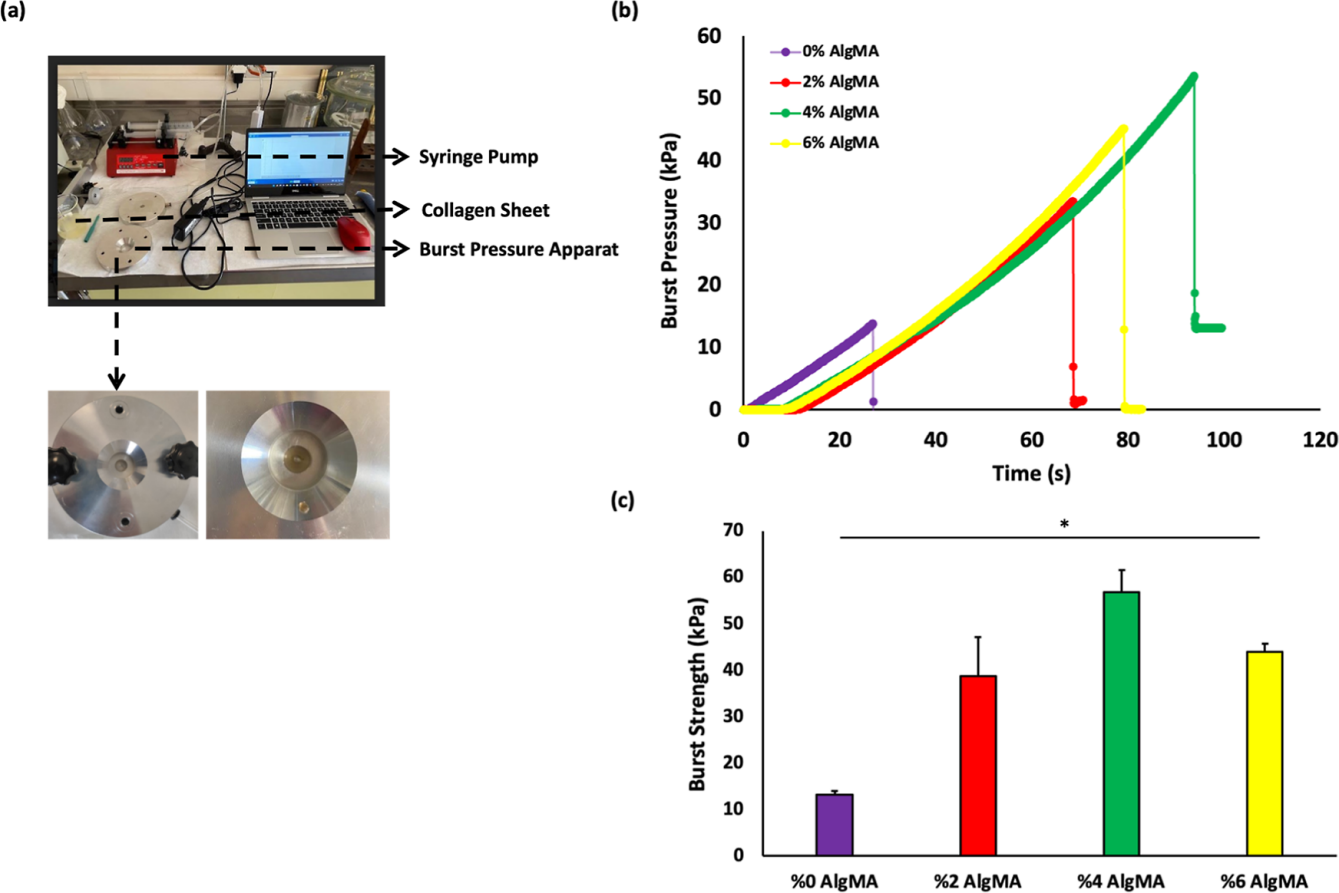
(a) Photos of Burst Pressure Apparatus. Experimental setup used for evaluating wound closure strength and precision-machined aluminum fixture featuring a 3 mm central opening to simulate tissue defects. (b) Burst pressure–time curves of different tissue adhesive hydrogels. (c) Burst Strength of different adhesive hydrogels (mean ± SD, *n* = 4).

The burst pressure experiment showed the adhesion performance between the material and the collagen layer, depending on the pressure profiles. Air was delivered to the burst pressure apparatus using a syringe pump, and the resulting pressure increased linearly until the adhesive material was damaged (Figure 3b). A sudden decrease in pressure occurs depending on the material (mechanical failure or delamination) behavior. The material underwent delamination when various concentrations of AlgMA were added.^27^

As shown in Figure 3b, the addition of AlgMA at different concentrations increased the adhesion interaction between the material and collagen layer. The increase in surface interactions was determined by the increase in the pressure profiles. The delamination time of AlgMA-added bioadhesives from the collagen layer also increased with increasing concentration. The burst pressures of the bioadhesive hydrogels containing 0% (w/v) AlgMA were determined as 13.2 ± 0.85 kPa. As the AlgMA concentration increased (2%, 4% and 6% (w/v)), the burst pressures were determined as 38.65 ± 8.41 kPa, 56.8 ± 4.81 kPa and 44 ± 1.69 kPa, respectively.

#### 3.4.2 Lap–Shear Test

The standard test method was applied to determine the strength properties of the adhesive hydrogels in lap-shear by tension loading. In particular, the mechanical properties of materials, particularly their adhesive characteristics, are critical parameters for assessing their suitability for practical applications. Therefore, in vitro applications are valuable in terms of quality control and surface suitability determination before ex vivo application, along with the mechanical properties of adhesion strength.

In this context, the in vitro lap-shear test was first performed. In this application, the shear strength of the material between two glass surfaces was determined by tensile testing. First, the glass surfaces were prepared by coating with gelatin (20% w/v). The adhesion strength of the bioadhesive on gelatin-coated surfaces was tested. The bioadhesive applied between the two glass surfaces was covalently and ionically cross-linked with visible light and Ca^2+^. Tension was then applied to the glass tips placed on the mechanical testing device. The cohesive and adhesion behaviors of the bioadhesive were determined on different surfaces (Figure 4a–c). The lap-shear of bioadhesive hydrogels containing 0% (w/v) AlgMA was determined as 6.2 ± 1.3 kPa. As the AlgMA concentration increased (2%, 4% and 6% (w/v)), the strengths were determined as 16.9 ± 0.4 kPa, 15 ± 5.7 kPa and 8.5 ± 2.1 kPa, respectively. The results showed similar in vitro sliding behavior of the bioadhesive hydrogels for all ratios. The in vitro adhesion strengths to the gelatin-coated glass surfaces did not differ significantly among the hydrogels.

**Figure 4:**
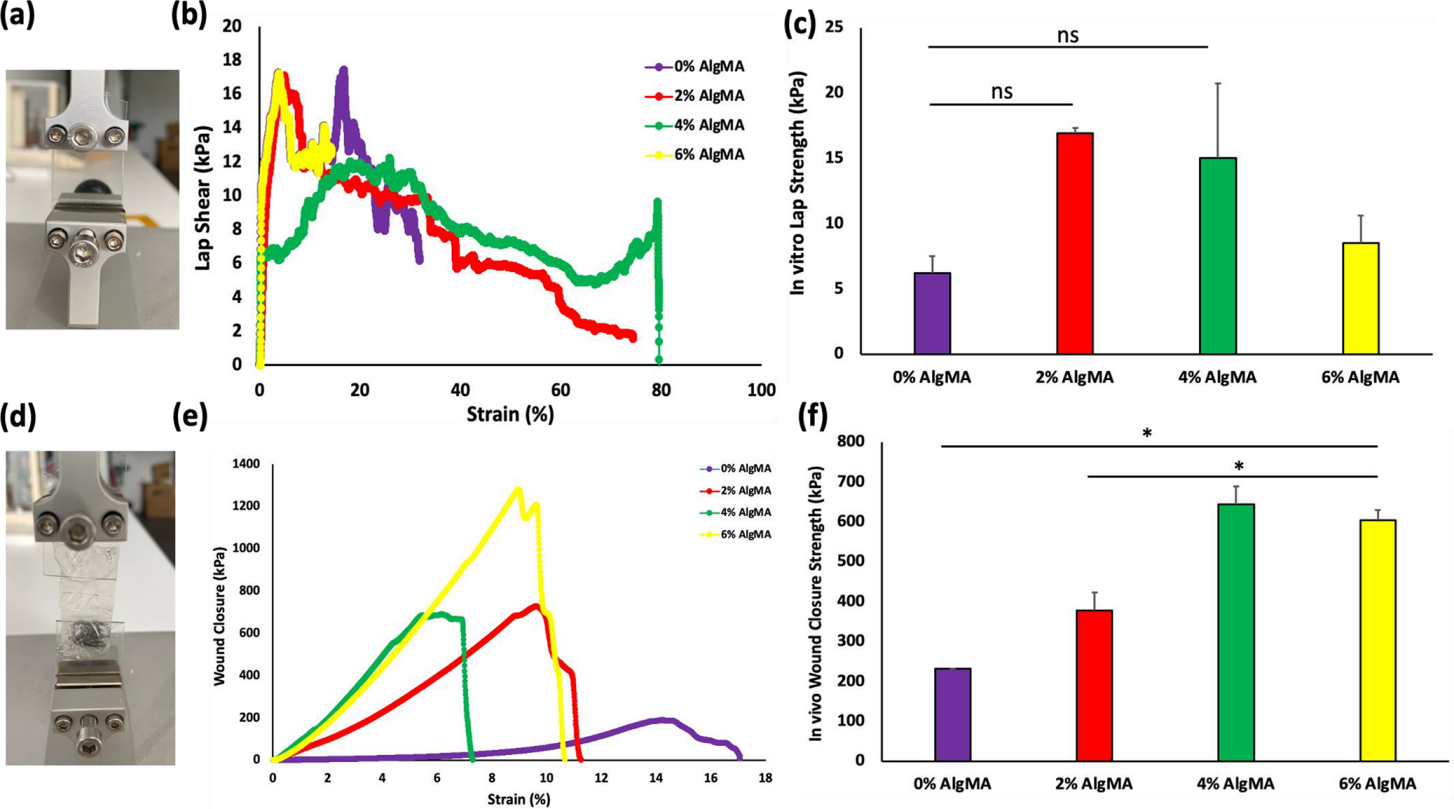
Photos of in vitro (a) lap shear and (d) wound closure test application. (b) Lap shear (kPa) strain (%) curves of different tissue-adhesive hydrogels. (c) Lap shear strengths of different adhesive hydrogels. (e) Wound closure (kPa)-strain (%) curves of different tissue-adhesive hydrogels. (f) Wound closure strength of the different adhesive hydrogels (mean ± SD, *n* = 4).

#### 3.4.3 Wound Closure Strength

The standard test method was applied to determine the wound closure strength. The resistance of the prepared bioadhesives to the applied mechanical forces and adhesive properties was tested to determine their suitability for use. A collagen sheet was prepared by separating the collagen sheet into two pieces. Bioadhesive materials were used to close these two pieces. Bioadhesive hydrogels were prepared by injecting them into the wound area in vitro, and then treating them with visible light and Ca^2+^. The areas where the collagen sheets were attached were placed in the device (Figure 4d), and tensile tests were performed. The test was completed by separating two collagen sheets from the wound area. Wound closure strength (kPa)–strain (%) elongation results were obtained, as shown in Figure 4e. The wound closure strength of bioadhesive hydrogels containing 0% (w/v) AlgMA was 231 ± 1.4 kPa. As the AlgMA concentration increased (2%, 4%, and 6% (w/v)), the strengths were 377 ± 46 kPa, 643.5 ± 46 kPa and 604.5 ± 24.7 kPa, respectively.

### 3.5 Ex Vivo Tissue-Sealing Characterization

#### 3.5.1 Burst Pressure

An ex vivo burst pressure test was performed using a cartilage sample obtained from the articular surface of the shoulder joint of a sheep. Specifically, hyaline cartilage (Cartilago hyalina) was carefully dissected from the glenoid cavity of the scapula and the head of the humerus. At the start of the test, damage (Figure 5a) was induced in the cartilage sample by using a punch to simulate a wound. The prepared tissue adhesive solution was then carefully applied to the damaged area, and after application, the adhesive was cross-linked, as in the in vitro application section.

**Figure 5:**
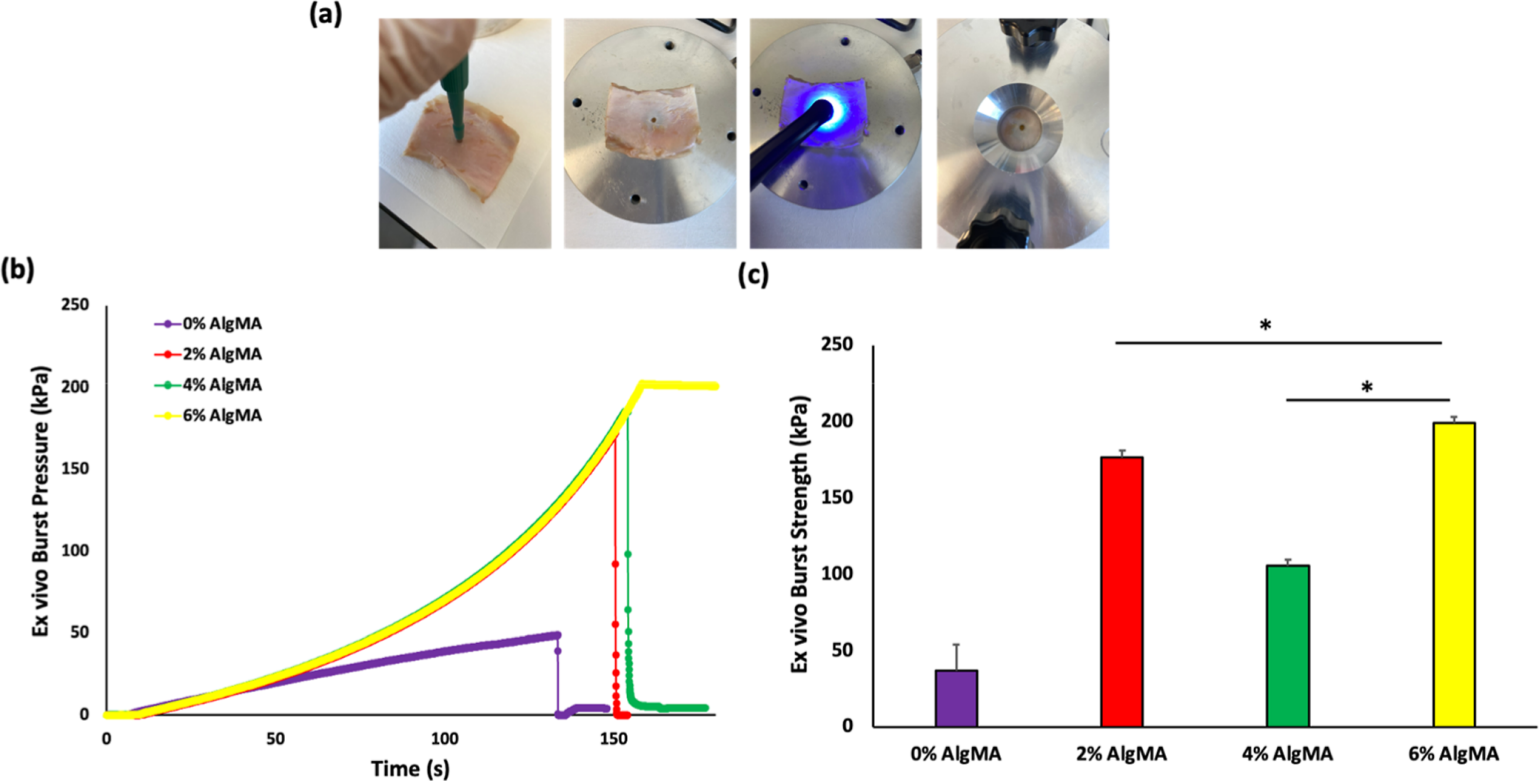
(a) Photographs of the ex vivo burst pressure test applications. (b) Burst pressure (kPa)––time (s) curves of different tissue-adhesive hydrogels. (c) Burst Strength of the different adhesive hydrogels (mean ± SD, *n* = 4).

The ex vivo burst pressure strength of bioadhesive hydrogels containing 0% (w/v) AlgMA was determined as 28.5 ± 18.6 kPa (Figure 5c). As the AlgMA concentration increased (2%, 4% and 6% (w/v)), the strengths were determined as 148 ± 48.5 kPa, 131.9 ± 46.2 kPa and 194.5 ± 8.5 kPa, respectively. The tissue adhesive containing 6% AlgMA is comparable to the Chondroitin Sulfate-PEG (CS-PEG)^42^ and GelMA + TG^43^ systems in the literature, with a burst pressure value of approximately 200 kPa. In another study which was published Hua et al., the burst pressure of DN hydrogels was 63.4 ± 4.6 kPa for HPC-Low gel and 78.7 ± 3.1 kPa for HPC-High gel, both above that of SN hydrogels, which recorded 10.4 ± 1.4 kPa for HAMA gel and 32.8 ± 2.8 kPa for HANB/GL gel.^24^

#### 3.5.2 Lap–Shear Test

The interfacial shear strength of the bonded tissues has long been a critical issue in tissue replacement or repair.^44^

The ex vivo lap-shear strength of the bioadhesive hydrogels containing 0% (w/v) AlgMA was determined as 295.5 ± 12.3 kPa. As the AlgMA concentration increased (2%, 4%, and 6% (w/v)), the strengths were determined as 335.5 ± 3.5 kPa, 277 ± 2.8 kPa and 384 ± 18.4 kPa, respectively (Figure 6c).

**Figure 6:**
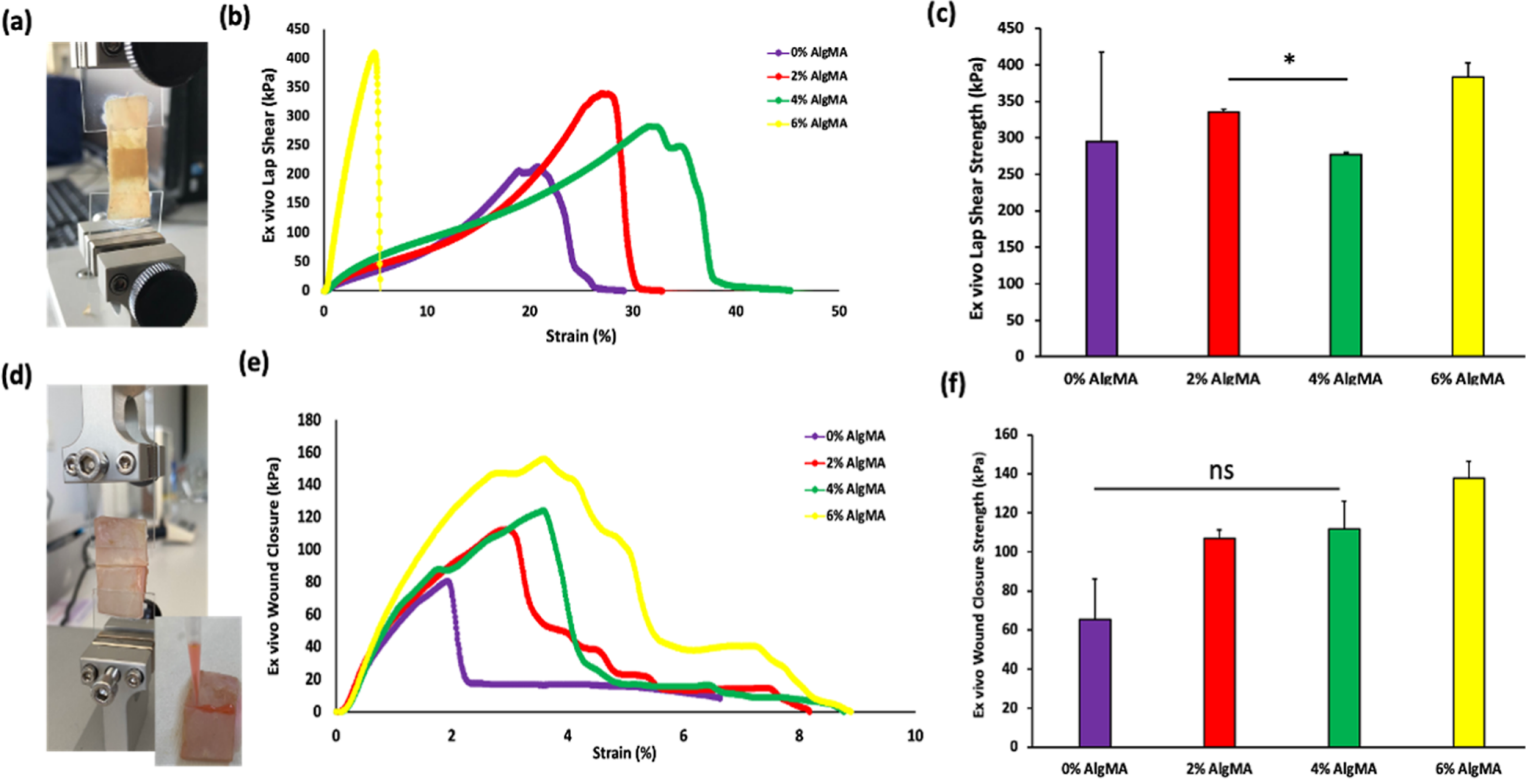
(a–d) Photographs of the cartilage adhesion measurement test of adhesion strength. (b) Lap shear stress (kPa)–strain (%) curves of different tissue-adhesive hydrogels. (c) Lap shear strengths of different adhesive hydrogels. (e) Wound closure stress (kPa)–strain (%) curves of different tissue adhesive hydrogels. (f) Wound closure strength of the different adhesive hydrogels (mean ± SD, *n* = 4).

Kazusa et al. evaluated the adhesive performance and cytocompatibility of LYDEX, an aldehyde-dextran and polylysine adhesive, when applied to the articular cartilage. The results indicated that LYDEX exhibited a mean adhesive strength of 15 kPa.^45^ In another study, Hua et al. introduced a novel hybrid photo-cross-linkable (HPC) hydrogel synthesized from hyaluronic acid (HA) and *O*-nitrobenzyl derivatives. This hydrogel demonstrated remarkable mechanical integrity, with tensile and shear strengths of 33.8 ± 4.6 and 47.4 ± 4.9 kPa, respectively, upon detachment from the cartilage surface.^24^ Similarly, Zhang et al. designed an advanced adhesive hydrogel by forming a cross-linked matrix of alginate–dopamine (AD), chondroitin sulfate (CS), and regenerated silk fibroin (RSF). The resulting AD/CS/RSF, hydrogel achieved a lap shear strength of 121.7 ± 12.3 kPa on wet substrates. A commercial enbucrilate (butyl cyanoacrylate) tissue adhesive was used as a control.^25^ Qiu et al., investigated the adhesive capacity of OHA/HTCCMA hydrogels by generating cartilage defects (diameter of about 5 mm) on porcine femoral heads. The O_8_H_2_ exhibited mean adhesion strengths of 0.72 and 1.49 kPa on healthy and degenerated cartilage, while the O_8_H_2_D variant displayed values 0.67 and 1.34 kPa, respectively.

#### 3.5.3 Wound Closure Strength

The ex vivo wound closure strength (Figure 6f) of bioadhesive hydrogels containing 0% (w/v) AlgMA was determined as 65.5 ± 20.5 kPa. As the AlgMA concentration increased to 2%, 4%, and 6% (w/v), the measured strengths rose to 107 ± 4.2 kPa, 112 ± 14.1 kPa, and 138 ± 8.5 kPa, respectively. These results indicate a clear positive correlation between AlgMA concentration and wound-sealing capability. As AlgMA concentration increased, the wound closure strength of the tissue adhesive increased significantly. This can be explained by densification of the network structure. The average sealing force also increased significantly with increasing AlgMA concentrations. The error bars indicate acceptable levels of repeatability. The 6% AlgMA group exhibited the highest force with the lowest variation. This indicates that 6% AlgMA showed the strongest adhesive performance, and the cross-link density probably reached the ideal level.

Increased concentrations of AlgMA increase the mechanical strength while preserving elasticity. This property may be advantageous, particularly in dynamic regions such as joints. To the best of our knowledge, no previous study has reported ex vivo wound closure strength measurements specifically on articular cartilage. Therefore, direct numerical comparison with literature values is not currently possible. However, the measured wound closure strength of 138 ± 8.5 kPa for the 6% (w/v) AlgMA hydrogel suggests a strong and consistent adhesive performance, especially considering the avascular and smooth characteristics of cartilage tissue, which typically hinder efficient sealing. The relatively low variation among replicates further supports the repeatability of the adhesion behavior under these challenging conditions. This study may therefore serve as an initial benchmark for ex vivo wound closure testing on cartilage substrates.

### 3.6 In Vitro Cytocompatibility Tests

LDH is a direct measure of cytotoxicity, indicating cell membrane damage. 3T3 cells were used for the time-dependent study of the samples. The results of the test conducted according to the criteria specified in the ISO10993–5 standard at three different time periods resulted in a minimum cell viability of 90.91% (control group, 72 h). In all groups, as shown in Figure 7, viability decreased slightly at 72 h, but this decrease was tolerable in terms of biocompatibility. Viability values were high (90–100%), which indicates low LDH release. However, a slight decrease was observed at 72 h. This may indicate a limited increase in membrane permeability, especially at 6% AlgMA, depending on cross-link density. However, these values are still far outside the cytotoxic range.

**Figure 7:**
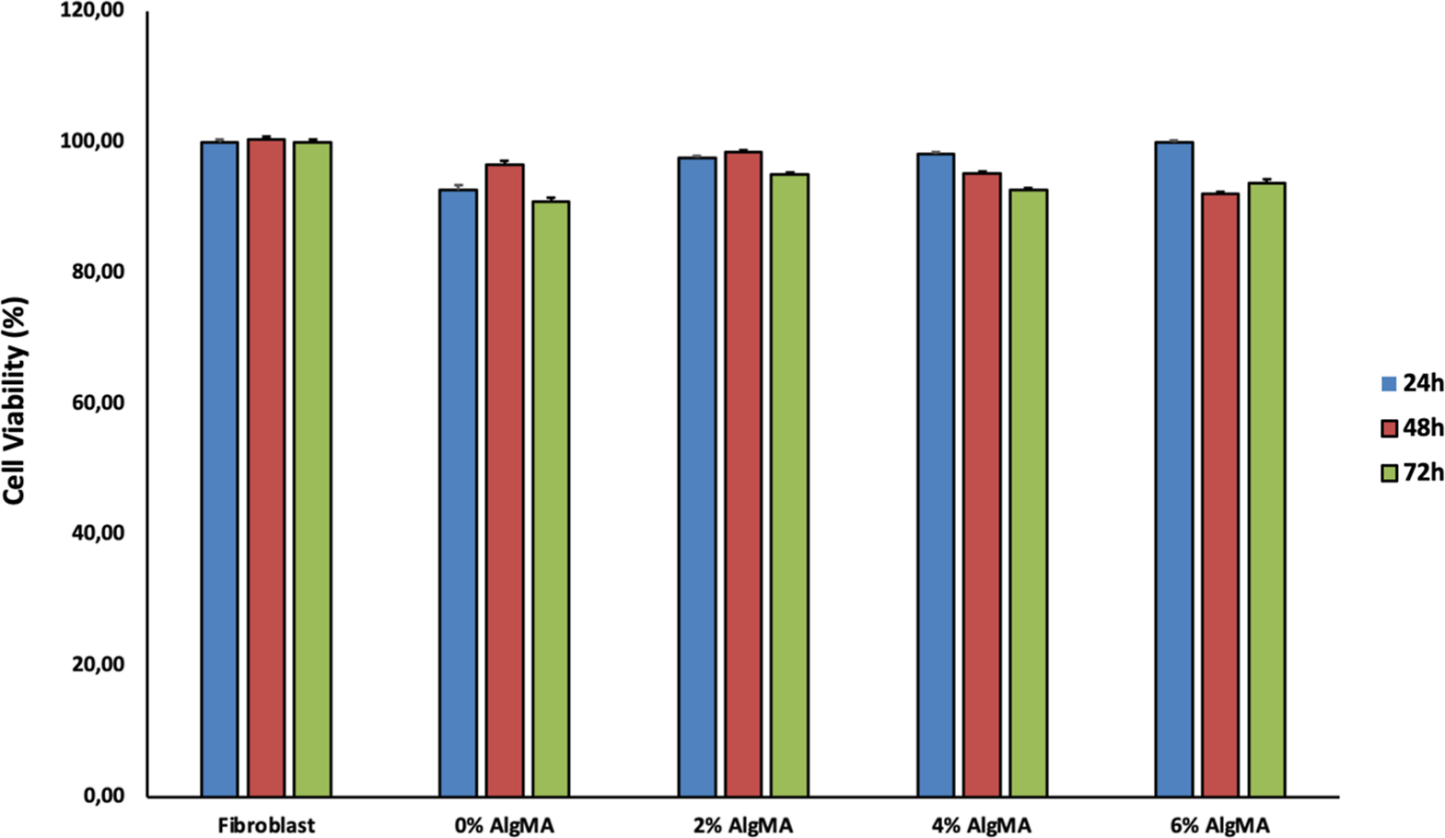
3T3 cell viability on differently coated surfaces for over 72 h. This is an image of cells on the third day.

The developed tissue adhesives showed better or equivalent performance than many reference adhesives in the literature in terms of both mechanical performance and biocompatibility. In particular, 2 and 4% AlgMA offered a balanced structure in terms of viability and stability.^43,46,47^ Since cell viability of 70% and above was determined as the biocompatibility limit in the ISO10993–5 standard protocol, all studied samples were shown to be biocompatible.

## 4 Conclusions

This study focused on the creation and comprehensive assessment of an innovative bioadhesive hydrogel for the repair of cartilage tissue. A sophisticated double-cross-linked hybrid hydrogel, comprising PEGDA and AlgMA, was created, capable of in situ cross-linking upon exposure to visible light and Ca^2+^ for the repair of cartilage lesions. The composite system demonstrated improved adhesion strength and biocompatibility in both in vitro and ex vivo environments, highlighting its potential as a substitute for current clinical adhesives. Regarding the second cross-linking mechanism (AlgMA-Ca^2+^ ionic gelation), this process is known to occur rapidly upon contact with calcium ions. In clinical scenarios, this can be achieved via dual-syringe systems or by pretreating the tissue surface with Ca^2+^ solution, both of which are documented methods in the literature. These strategies support the feasibility of the hydrogel system for in situ applications.

These findings augment current research on tissue adhesives and facilitate the progression of individualized regenerative therapies. Although potential interactions between methacrylate moieties and nucleophilic groups (e.g., thiols or amines) on tissue surfaces are proposed, these mechanisms remain hypothetical in this study and require further experimental confirmation. Future research will focus on the long-term in vivo efficacy and integration of bioactive compounds to enhance healing efficiency.
